# North Atlantic Ocean Circulation and Decadal Sea Level Change During the Altimetry Era

**DOI:** 10.1038/s41598-018-37603-6

**Published:** 2019-01-31

**Authors:** Léon Chafik, Jan Even Øie Nilsen, Sönke Dangendorf, Gilles Reverdin, Thomas Frederikse

**Affiliations:** 1 Department of Meteorology and Bolin Centre for Climate Research, Stockholm University, Stockholm, Sweden; 2 Geophysical Institute, University of Bergen, and Bjerknes Centre for Climate Research, Bergen, Norway; 3 Nansen Environmental and Remote Sensing Center, and Bjerknes Centre for Climate Research, Bergen, Norway; 4 Research Institute for Water and Environment, University of Siegen, Siegen, Germany; 5 LOCEAN/IPSL, Sorbonne Université/CNRS/IRD/MNHN, Paris, France; 6 Jet Propulsion Laboratory, California Institute of Technology, Pasadena, California USA

## Abstract

Regional sea-level rise is characterized by decadal acceleration and deceleration periods that typically stem from oceanic climate variability. Here, we investigate decadal sea-level trends during the altimetry era and pin down the associated ocean circulation changes. We find that decadal subpolar gyre cooling (warming), strengthening (weakening), widening (shrinking) since the mid-2000s (early 1990s) resulted in negative (positive) sea level trends of −7.1 mm/yr ± 1.3 mm/yr (3.9 mm/yr ± 1.5 mm/yr). These large-scale changes further coincide with steric sea-level trends, and are driven by decadal-scale ocean circulation variability. Sea level on the European shelf, however, is found to correlate well with along-slope winds (R = 0.78), suggesting it plays a central role in driving the associated low-frequency dynamic sea level variability. Furthermore, when the North Atlantic is in a cooling (warming) period, the winds along the eastern boundary are predominantly from the North (South), which jointly drive a slowdown (rapid increase) in shelf and coastal sea level rise. Understanding the mechanisms that produce these connections may be critical for interpreting future regional sea-level trends.

## Introduction

Sea level rise poses a major threat to the coastal societies and environment^1^. Global sea-level rise has been observed to accelerate synchronously with, and as a consequence of, anthropogenic global warming^2^. In fact, most recent studies^3^ show that global mean sea level rise has been accelerating from 1–2 mm/yr before 1990 to unprecedented high rates of about 3 mm/yr thereafter (Fig. S1). This acceleration is primarily a contribution from ice melting and ocean thermal expansion^3^. On top of the general global mean sea level rise, there are indeed decadal acceleration and deceleration periods^4–7^, especially on regional scales, which likely stem from basin-scale decadal ocean circulation changes^8–13^.

Oceanic climate variability in the North Atlantic is known to be dominated by decadal-to-multidecadal fluctuations^14,15^ that have profound regional and global climate impacts^16,17^. Recent observational evidence^18^ shows that the strength of the Atlantic Meridional Overturning Circulation or AMOC^19^, i.e. the flow of warm surface waters polewards and the return of cold deep waters equatorwards, is indeed the major factor regulating the warm and cold decades of the North Atlantic as has long been hypothesized^20^. This decadal-scale AMOC variability can have substantial influence on dynamical sea-level change, especially on regional scale, as a result of variable amount of mass, heat and freshwater redistributed by ocean currents.

Strengthening or weakening of ocean currents can induce substantial dynamical and steric regional sea-level changes^5,21,22^. In the North Atlantic, numerous studies have shown the marked increase in sea level over the Subpolar North Atlantic (SPNA), a key climatic region where the maximum node of the Atlantic multidecadal variability is found^23–25^, and associated spin-down of the gyre circulation to be a response to a decrease in wind stress curl over the region during mid-1990s^26,27^. This dynamic sea level change was also influenced by reorganization of the gyre boundaries that redistributed water masses, mostly of subtropical origin, around the rim of the gyre^27–29^ in the northern North Atlantic leading to a significant decadal change in density and hence steric sea-level rise. However, a recent study reports that this SPNA warming led to a reduction of the deep-water formation rate in the Labrador Sea, which led to an anomalously weak poleward heat transport and thereby triggering a cooling trend since around 2005 in the SPNA^14^. This is supported by direct observations from the RAPID array at 26°N, which have evinced an AMOC decline of about −0.3 Sv/yr between 2004 and 2015, likely as a recovery from the previous spin-up of the AMOC and mid-1990s SPNA warming^30^.

Because of the pronounced natural decadal climate variability of the North Atlantic induced by the overturning circulation, and the fact that the relatively short period of the satellite altimetry (since 1993) as compared to the emergence time of a forced linear trend signal^31,32^, it may not be suitable to study the long-term spatial sea-level trend patterns and relationship to the AMOC state (although the observed pattern may be similar to that projected by climate models as a response to a persistent AMOC slowdown^33^, cf. Fig. S2). Instead, we focus on quantifying the spatio-temporal patterns of decadal sea-level change in the North Atlantic consistently for the warming period (WP; 1993–2004) and cooling period (CP; 2005–2016), and investigate to which extent physical processes related to ocean circulation and wind changes explain the observed patterns. Lastly, we investigate the drivers of dynamic sea-level variability on the east Atlantic shelf and discuss how it may possibly relate to the observed decadal North Atlantic Ocean variability. This possible relationship is motivated by studies that showed a close link between steric height changes in the North Atlantic and sea-level variability in the eastern Atlantic along the European shelf and coast up to the Barents Sea^34–39^.

## Results and Discussion

### Dynamic and steric sea level trends

**Figure 1:**
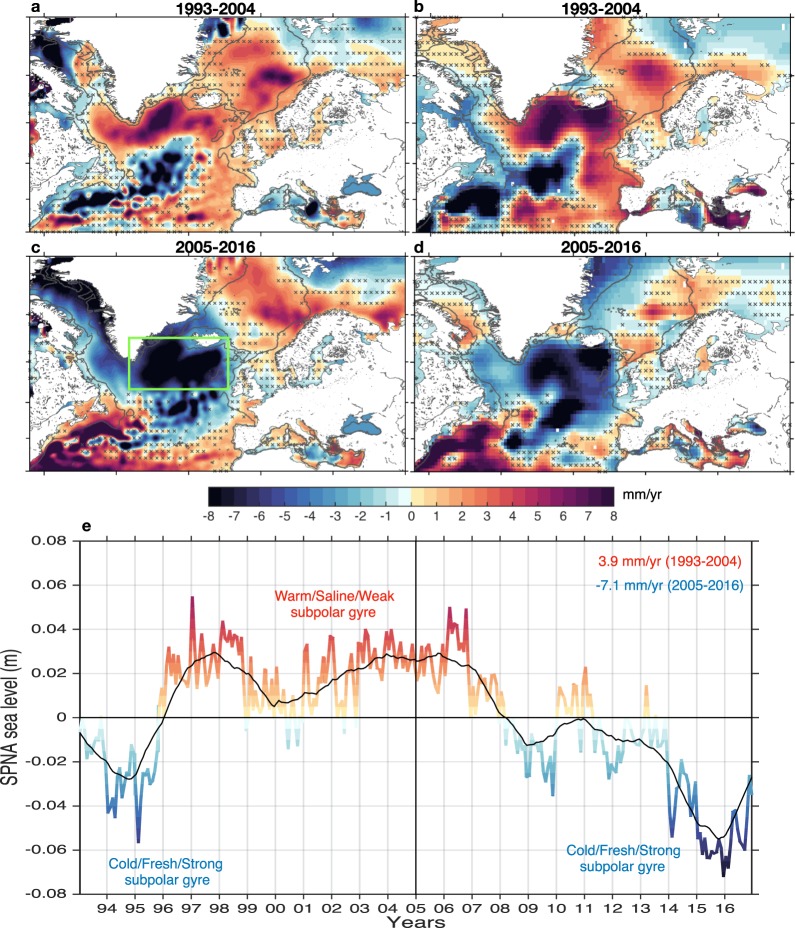
Regional dynamic and steric sea level trends in the North Atlantic Ocean. The dynamic and steric sea level trends (mm/yr) as deduced from altimetry^68^ and EN4 hydrographic dataset^69^, respectively, for the (**a**,**b**) 1993–2004 and (**c**,**d**) 2005–2016 periods. The global mean sea-level and steric height trend of each period have been removed before calculating the regional trends. The stipplings indicate the non-significant regions at the 95% confidence level using the modified Mann-Kendall test^80^. The gray contours in all panels depict the 500 and 1000-m isobaths. (**e**) The averaged monthly dynamic sea level in the SPNA [45°W–5°W, 55°N–65°N] (see box in panel c) as calculated from altimetry. The global mean sea level (Fig. S1) has been removed beforehand. The black line is the smoothed SPNA sea level using a 25-month running mean in order to represent interannual-to-decadal timescales. Anomalously high/low sea levels do not only represent warming/cooling of the upper ocean but also the strength of the subpolar gyre^45^. The figure was produced using the software Matlab R2014b, (https://www.mathworks.com).

Changes in dynamic and steric sea level from altimetry show a significant upward trend in the eastern North Atlantic, the subpolar gyre and interior Nordic Seas during the WP (Fig. 1a,b). In contrast, these trends are reversed in the mid-latitudes and over the Gulf Stream (GS) region. During the CP, however, this SPNA-GS sea-level dipole trend pattern is opposite to that of the WP (Fig. 1c,d). In general, the North Atlantic shows a coherent horse-shoe-shaped sea level signal, which has a strong upward trend during the WP and a decline during the CP, suggesting a key role for ocean dynamics^40,41^. This decadal-scale anti-phase relationship between the SPNA and the GS region projects well on the observed decadal AMOC fingerprint^42,43^, which has seen a decline since mid-2000s (Fig. S3). In addition, the close resemblance between dynamic sea level from altimetry and steric height trends suggest that a significant source of this decadal sea level change is predominantly of steric origin.

This decadal-scale rise and fall of SPNA sea level (Fig. 1e) further reflects the strength and shape of the wind-driven subpolar gyre system^26–28,44,45^, which is considered to be a qualitative measure of the AMOC^42,46,47^. Anomalously higher (lower) sea levels in the SPNA coincide with weaker (stronger) as well as a spatially contracted (expanded) subpolar gyre. This contraction-expansion of the subpolar gyre is important as it regulates the inflow of warmer and saline subtropical waters into the northeast Atlantic, subpolar gyre and the Nordic Seas^26–28,48^. Figure 1e indicates that the subpolar gyre was strong between 1993 and 1995, and thereafter anomalously weak for about a decade, i.e. between the mid-1990s and mid-2000s. After 2005, we observe a gradual transition from a weak to a strong subpolar gyre, which is related to the cooling and freshening trend of the SPNA. The anomalously low sea level during the past few winters (2014–2016) can be attributed to the exceptionally strong North Atlantic Oscillation^49^ and hence a return to the conditions seen during the early 1990s. We estimate the regional SPNA trend during the WP and CP to be about 3.9 ± 1.5 mm/yr and −7.1 ± 1.3 mm/yr, respectively.

The SPNA-GS decadal sea-level trends are characterized by a dipole pattern (Fig. S3), which projects onto the AMOC fingerprint^42,43^ (shifted backward by 4 years; see methods), hereby indicating a key role for ocean circulation. The observed trend pattern arises partly due to different heat-advection speeds along the western boundary as discussed by Zhang and Zhang^43^. A positive (negative) AMOC anomaly at higher latitudes induces a convergence/divergence (divergence/convergence) of the meridional heat-transport anomaly in the SPNA/GS region, which leads to a SPNA warming (cooling) and a cooling (warming) in the GS region some years after^43^. However, it can also be triggered by changes in the convection and deep-water formation in the Labrador Sea, as proposed by Robson *et al*.^50^. They suggest that this mechanism may have played a key role for the evolution of the subpolar gyre regime shifts through reduced heat convergence^51,52^. For the CP, there is evidence from direct measurements of the RAPID array and ocean reanalysis (GloSea5) that the AMOC has seen a decline since mid-2000s of about −0.30 Sv/yr^30^, which followed an increase since the mid-1990s. This suggests that the decadal AMOC variability shows compatible trends with the decadal sea level reversals of the subpolar gyre, which may be in line with the results reported by ref.^53^. Previous work has, in fact, shown that initializing models from a strong AMOC state^54^ is crucial to capture the observed decadal warming and weakening of the subpolar gyre (see also refs^44,55^). However, trends in the strength of the wind-stress curl further suggest a role for Sverdrup dynamics as will be discussed in the context of decadal changes in the next section.

### Atmospheric forcing and shifting ocean currents

**Figure 2:**
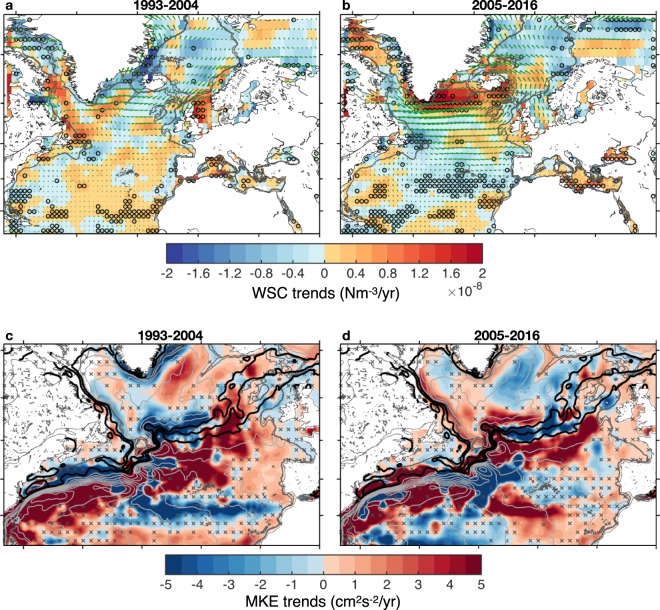
Atmospheric-driven ocean changes. Trends of wind-stress curl (WSC) calculated using NCEP/NCAR reanalysis for (**a**) the 1993–2004 WP and (**b**) the 2005–2016 CP. The vectors in the upper panels represent the wind-stress trends. The circles in the upper panels indicate the regions that are significant at the 95% confidence level^80^. Trends of mean kinetic energy (MKE) calculated using altimetry for (**c**) the 1993–2004 WP and (**d**) the 2005–2016 CP. The stipplings indicate the non-significant regions at the 95% confidence level. The light grey contours depict the time-invariant mean dynamic topography (CNES-CLS2013 MDT) that ranges from 0.8 m to −0.8 m with a spacing of 0.1 m. Three of these contours (−0.1, −0.2 and −0.3 m) are highlighted in black as they trace the different branches of the NAC^61^ that continue into the northeast Atlantic. The gray contours in all panels depict the 500 and 1000-m isobaths. The figure was produced using the software Matlab R2014b, (https://www.mathworks.com).

We now investigate decadal changes of the wind-stress curl over the North Atlantic Ocean. During the WP, we do not observe any significant wind-stress curl trends in the North Atlantic region (Fig. 2a). The midlatitude wind-stress anomalies in the WP are weak and directed eastward, i.e. opposing the mean westerly wind direction. While during the CP, a significantly pronounced increase of the wind-stress curl over the SPNA can be observed (Fig. 2b). The wind-stress anomalies display a strong zonal wind-stress increase over the subpolar-subtropical gyre boundary, as well as a strong cyclonic wind-stress increase over the SPNA. The fast barotropic response to the observed wind-stress curl trends during the CP produces a high to low sea level transition and hence a weak to strong subpolar gyre conditions. However, the question arises whether there has been any shift in the North Atlantic ocean surface currents?

To detect shifts in the main surface pathways, we look for decadal increase or decrease in the Mean Kinetic Energy (MKE) calculated from altimetric absolute geostrophic surface velocities (see methods). Changes in the kinetic energy can be interpreted as spatial shifts in the paths as well as the intensity of the currents^56–60^. During the WP, the GS is seen to have migrated southwards as indicated by the MKE increase south of its mean position as depicted by the mean dynamic topography contours (Fig. 2c). The MKE further shows a general increase east of the Mid-Atlantic Ridge extending into the eastern SPNA (in a tilted fashion), around the Iceland Basin and the Irminger Sea tracing the main current branches^61^. The currents along the western boundary in the SPNA show a negative trend that is consistent with a decline of the subpolar gyre strength^26^. During the CP, however, the MKE experiences a negative trend in the northern North Atlantic, and a positive trend that extends more to the east in a zonal manner east of the Mid-Atlantic Ridge along the path of the North Atlantic Current (NAC), signifying an overall southeast shift of the NAC and subpolar gyre (Fig. 2d). In addition, the East Greenland and Labrador boundary currents show a strengthening during the CP, which is further indicative of a recovery of the subpolar gyre from earlier weakening^45^, and the GS seems closer to its mean position during the CP. These changes are summarized in Fig. S4.

The kinetic-energy results suggest that the recent decadal cooling of the SPNA coincided with a strengthening as well as a spatial expansion of the subpolar gyre as demonstrated through the southeastward shift of the NAC. This frontal structure change during the CP is a response to a more zonal orientation of the zero wind-stress curl (Fig. 2b) inducing an expansion of the subpolar gyre and hence a reduction in the access of warm and saline subtropical waters into the subpolar gyre through the northeast Atlantic (Figs S5 and S6), a key region where ocean advection is found to dominate^62^. These results emphasize the role played by the spatial extent of the gyre in generating the SPNA climatic shifts that in turn influence the decadal sea level variability. This view on the importance of the horizontal extent of the gyre is in close agreement with the recent study of Piecuch *et al*. (2017), though they merely focused on the local wind-stress curl at the subtropical-subpolar gyre boundary^63^ as an instigator of the decadal SPNA climatic reversals. This is different from the buoyancy-driven response suggested by Robson *et al*.^50^ to have triggered the cooling trend of the SPNA since 2005, but these authors mention that the relative roles of midlatitude wind-stress curl and buoyancy forcing for the observed changes are poorly understood. It is very likely that both mechanisms suggested by Piecuch *et al*. (2017) and Robson *et al*.^50^ have played equal roles (C. Piecuch, personal communication).

### Shelf response to wind forcing

Recent studies show that coastal sea level along the European coast project onto a basin-wide horse-shoe-shaped steric pattern^37,38^ similar to Fig. 1. This pattern also projects onto the decadal AMOC variability, which suggests that enhanced (reduced) poleward heat transport and subtropical waters into the northeast North Atlantic (Fig. S6) may coincide with an acceleration (slowdown) of sea level rise on the northern European shelves and coasts^64^. However, for the eastern boundary to adjust, cross-shelf heat flux due to Ekman transport (and upwelling/downwelling processes) is required since it is the mechanism by which the observed oceanic horse-shoe-shaped steric signal can be steered onto the shelf and influence the dynamic sea level. Therefore, it is reasonable to investigate how along-shelf winds (see methods) have varied during the altimetry period.

**Figure 3:**
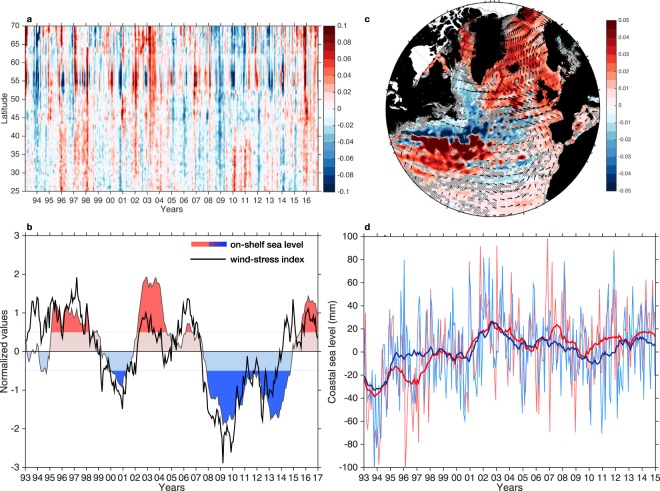
Low-frequency dynamic shelf sea level variability. (**a**) Time-latitude diagram of regional dynamic shelf sea level variability in the eastern Atlantic with no smoothing applied. (**b**) Averaged regional dynamic sea-level variability over the east Atlantic shelf (shallower than the 500-m isobath) calculated from altimetry for the 1993–2016 period (shading) overlaid by the latitudinally-averaged along-slope wind-stress (black). The time series have been deseasoned, smoothed with a 25-month running mean and normalized. Dark blue and red indicate anomalous periods (higher and lower than 0.5 standard deviation). (**c**) Composite analysis of sea level (shading) and wind-stress (vectors) calculated based on the difference between the anomalously high and low dynamic shelf sea-level periods shown in panel (a) but excluding the first and last year due to smoothing effects. (**d**) Monthly (thin lines) and smoothed (thick lines) relative sea-level variability from tide gauges in the North (red) and Norwegian (blue) Seas. The figure was produced using the software Matlab R2014b, (https://www.mathworks.com).

Figure 3 demonstrates the nature of dynamic sea-level fluctuations on the eastern Atlantic shelf and how they relate to along-slope wind-stress variability. We find that sea level on the shelf from the subtropics to higher latitudes exhibits spatially coherent variations on multiple timescales ranging from monthly to decadal, Fig. 3a. On low-frequency timescales, most of the dynamic shelf sea level variability along the eastern boundary can evidently be explained by along-slope wind-stress variability (*R*^2^ = 61%), Fig. 3b, as reinforced by the composite analysis in Fig. 3c, where anomalously higher (lower) dynamic shelf sea-level periods coincide with higher (lower) sea level on the entire European shelf, SPNA and Nordic Seas, and winds generally directed poleward (equatorward) along the continental slope. Thus, the observed meridional winds and associated cross-shelf Ekman transport play a major role in forcing the dynamic shelf sea-level variability on interannual-to-decadal timescales in the eastern Atlantic from the subtropics all the way to the Barents Sea. It is, however, striking that in general southerly (northerly) winds are more common when the North Atlantic is undergoing a warming (cooling) in the context of decadal-to-multidecadal variability, but also the fact that northerly winds during the CP persisted for a remarkable period of 7 years (2008–2014).

Anomalously positive (negative) along-slope winds and thus higher (lower) dynamic sea level periods on the east Atlantic shelf further appear to synchronize a pattern consisting of anomalously higher and lower sea levels south and north of the GS path, respectively, as shown in the composite analysis (Fig. 3c). This distinctive pattern in the subtropical western Atlantic of a southerly (northerly) GS position is indicative of a strong (weak) northern recirculation gyre^65^ as well as a strong (weak) western boundary current consistent with a strong (weak) phase of the AMOC^66^. It is thus reasonable to suggest that a stronger (weaker) heat transport induced by AMOC variability during the WP (CP) produced a steric sea-level increase (decrease) that is communicated to the shelf with the aid of along-slope winds; an efficient driver of cross-shelf exchange. Altogether, these results further highlight the importance of the combined low-frequency along-slope wind-stress variability and oceanic steric forcing in modulating the rate of decadal coastal sea-level rise in the North and Norwegian Seas as demonstrated in Fig. 3d. A notable aspect is the observed decadal-scale acceleration and slowdown (or flattening) in coastal sea-level rise before and after 2004, respectively.

## Summary and Conclusions

In this study we have investigated the decadal rates of change of the warming and the cooling periods over the North Atlantic Ocean during the satellite altimetry era. We have not assessed the long-term (1993–2016) trends since the decadal variability in the North Atlantic is strong and the time of emergence of a forced signal is longer than the period of the altimetry^31,32^. The first main message conveyed is that decadal sea level change from altimetry over the open ocean, in particular over the SPNA, is driven by steric changes. The SPNA warmed/salinified and cooled/freshened in the first/second 12-year period of satellite altimetry. Concurrent with these changes, the subpolar gyre weakened and strengthened significantly during the WP and CP of altimetry, respectively. These decadal sea level changes are further consistent with recent studies demonstrating that the AMOC exhibits similar decadal-scale variability^18,30^.

We observe that a strong (weak) subpolar gyre during the CP (WP) is associated with a strengthened (weakened) wind-stress curl, a horizontally expanded (contracted) gyre, a southeastwards (northwestwards) shifted NAC pathway. These results support the notion of reduced (increased) advection of warm and saline subtropical waters into the northeast Atlantic and a role for Sverdrup dynamics. These cold (warm) and fresh (saline) periods lead to a lower (higher) steric anomaly that communicates with the east Atlantic shelf through along-slope winds (via Ekman transport and upwelling/downwelling processes), which play a major role in producing the interannual-to-decadal dynamic shelf sea-level variability in the east Atlantic and in modulating the pace of decadal coastal sea level rise in the North and Norwegian Seas.

In a way, we have in this study decomposed the correlation pattern between steric height and coastal sea level shown by Dangendorf *et al*.^37^ and Frederikse *et al*.^38^, explained its associated spatio-temporal variability and revealed that it emerges as a result of a complex combination of oceanic processes involving overturning and gyre-scale circulation changes as well as along-slope winds. It is, however, remarkable that the low-frequency along-slope wind variability conducive of a cross-shelf transport and coherent dynamic sea-level change on the shelf in the eastern Atlantic is in synchrony with the observed ocean circulation changes. It is also possible that the climatic reversals of the North Atlantic are associated with atmospheric patterns favourable of anomalous along-slope winds that promote the ocean-to-shelf connection. Thus, capturing the combined dynamical forcing mechanisms pointed out in this study may be critical for representing realistic decadal-scale rise and slowdown periods in future sea-level projections along the eastern Atlantic coast, especially for the European shelves and coasts.

Finally, we suggest that our results may have implications for decadal European sea-level predictions based on the climate state of the North Atlantic Ocean, which have been shown to exhibit substantial skill in the SPNA^62^. However, the state of the atmosphere may well be equally important as that of the ocean. This is particularly true during cold North Atlantic Ocean periods, which are found to alter the baroclinicity of the atmosphere thereby leading to a positive phase of the North Atlantic Oscillation^67^, as occurred during the past few winters. During this recent period, European shelf and coastal sea levels increased rapidly (e.g. Fig. 3b) as a response to the strong westerly winds associated with the North Atlantic Oscillation, despite an anomalously cold North Atlantic Ocean.

## Methods

### Satellite altimetry

The DUACS DT2014 multi-mission satellite altimetry^68^ is here utilized to study the sea-level trends and variability in the North Atlantic and on the European shelves. We use the 1/4° degree monthly mean absolute dynamic topography (the sum of sea-level anomalies and the MDT_CNES/CLS 2013 ocean mean dynamic topography) averaged from daily data between January 1993 and December 2016. The sea-level anomalies in DT2014 are referenced to the 1993–2012 period. Geophysical corrections such as tides, dry/wet tropospheric and inverse-barometer effects have been applied beforehand. The sea level anomalies have been reprocessed and as a result significant improvements in, for example, surface geostrophic currents, mesoscale eddies and their kinetic energy, as well as sea level at higher latitudes are demonstrated^68^. Sea levels at coastal areas have also been improved and they show good consistency when compared to tide gauges. The results pertaining to regional sea level trends over the North Atlantic Ocean were calculated by subtracting the global mean sea-level trend (cf. Fig. S1) from altimetry estimated for the specific period under investigation (see e.g.^11^). The altimetry data in this study have been used to calculate sea level and mean kinetic energy trends (MKE). The MKE is defined as:

1a $$MKE=\frac{1}{2}({u}_{g}^{2}+{v}_{g}^{2}),$$

where the absolute geostrophic velocities are defined as:

1b $${u}_{g}=-\,\frac{g}{f}\frac{\partial \eta }{\partial y},$$

1c $${v}_{g}=\frac{g}{f}\frac{\partial \eta }{\partial x}.$$

here *η* is the absolute dynamic topograhpy, *u*_*g*_ and *v*_*g*_ are the zonal and meridional absolute geostrophic velocities, *g* the gravitational acceleration, and *f* the Coriolis parameter.

### Steric height data and calculation

The temperature and salinity data are based on the monthly mean objectively analyzed hydrographic data from the UK Met Office, EN4v2^69^, with the bias taken into account using the correction by Gouretski and Resghetti (2010)^70^. We use the 1993–2016 period to calculate *in-situ* density using the non-linear equation of state^71^, and later integrate the density departures from the time-mean (1993–2016) over the upper 1000 m of the water column to derive the steric height:

1d $$\eta =-\,\frac{1}{{\rho }_{0}}{\int }_{-H}^{0}(\rho -\bar{\rho })dz,$$

where *ρ*_0_ is the reference density (1025 *kg*/*m*^3^), *H* is the reference depth, *ρ* is the *in situ* density, *z* denotes depth, and $\bar{\rho }$ is the time-mean density. The results pertaining to regional steric-height trends over the North Atlantic Ocean were calculated by subtracting the global mean steric-height trend from EN4v2 (66°S–66°N) for the specified period.

### AMOC fingerprint

The ‘AMOC fingerprint’^42^ time series is the first principal component of the subsurface temperature field at 400 m [80°W–0°W, 20°N–65°N] from the EN4v2 dataset^69^ calculated using Empirical Orthogonal Functions^72^ and explains 19% of the variance (see Fig. S3). To better represent AMOC anomalies at our focus region, i.e. at middle and higher latitudes, the AMOC fingerprint is shifted 4 years back in time^73^ due to the slow advection speed^43^ of the related anomalies along the western boundary that induces the observed horse-shoe pattern (Figs 1 and S3) most distinguished by the anti-phase relationship between the SPNA and the GS. The mechanism for the evolution of the AMOC fingerprint can be summarized as follows: A positive (negative) AMOC anomaly at higher latitudes leads to a convergence/divergence (divergence/convergence) of the meridional heat transport anomaly in the SPNA/GS region, which leads to a SPNA warming (cooling) and a cooling (warming) in the GS region some years after^43^.

### Atmospheric renalysis

For the atmospheric circulation analysis, we use the NCEP/NCAR reanalysis^74^ monthly zonal and meridional winds and wind stress spanning the 1993–2016 period. The horizontal resolution of these data is 2.5° × 2.5°. The data have been deseasonalized by removing the mean seasonal cycle prior to the analysis. The wind-stress data are further used to calculate the wind stress curl in the North Atlantic region. A comparison of the NCEP/NCAR wind-stress curl trends and those derived from ERA-interim^75^ are found to be compatible (cf. Fig. S7).

### Wind-stress index

To investigate the role played by wind stress on the northern European shelf, we spatially average the sea level on the shelves and construct an index of its variability on interannual-to-decadal timescales using a 25-month moving average following^39^. Furthermore, by rotating the zonal and meridional wind-stress components to follow the continental slope, i.e. tracing the 500-m isobath, and averaging along this contour we construct an along-slope wind-stress index for the 1993–2016 period, which is proportional to the zonal component of the Ekman transport.

### Coastal sea level

To obtain an estimate of regional coastal sea-level changes, we use tide-gauge data from the Permanent Service for Mean Sea Level or PSMSL^76^. We use all stations that have at least 20 years of data over the 1993–2014 period and who are not flagged in the PSMSL database. Since we are interested in large-scale ocean dynamic signals, the effects of GIA^77^, nodal cycle, and present-day mass redistribution have been removed from each individual tide-gauge record, as well as the response to local pressure and wind effects^78^, see ref.^38^ for a description of the used models and methods.

To merge the individual corrected tide-gauge records into a region-mean estimate, the virtual-station method^3,79^ has been applied. In this method, the two closest stations are subsequently merged into a new virtual station halfway between both stations. This procedure is repeated until only one virtual station is left, which is used as the region-mean estimate. We require a 20-year overlap between the stations to remove the common mean height over that period.

## Supplementary information

Supplementary info
