# Supplementary material for: North Atlantic Ocean Circulation and Decadal Sea Level Change During the Altimetry Era
Source: Sci Rep. 2019 Jan 31;9:1041. doi: 10.1038/s41598-018-37603-6 (PMC6355806; doi:10.1038/s41598-018-37603-6)
Supplement: Supplementary file 1 — Supplementary info [file 41598_2018_37603_MOESM1_ESM.pdf]

Supplementary information for

North Atlantic Ocean circulation and decadal sea level change during the altimetry era

Chafik L., Nilsen J. E. Ø, Dangendorf S., Reverdin G. and Frederikse T.

correspondence to: leon.chafik@misu.su.se

This PDF file includes:

1. Supplementary Figures S1 to S7

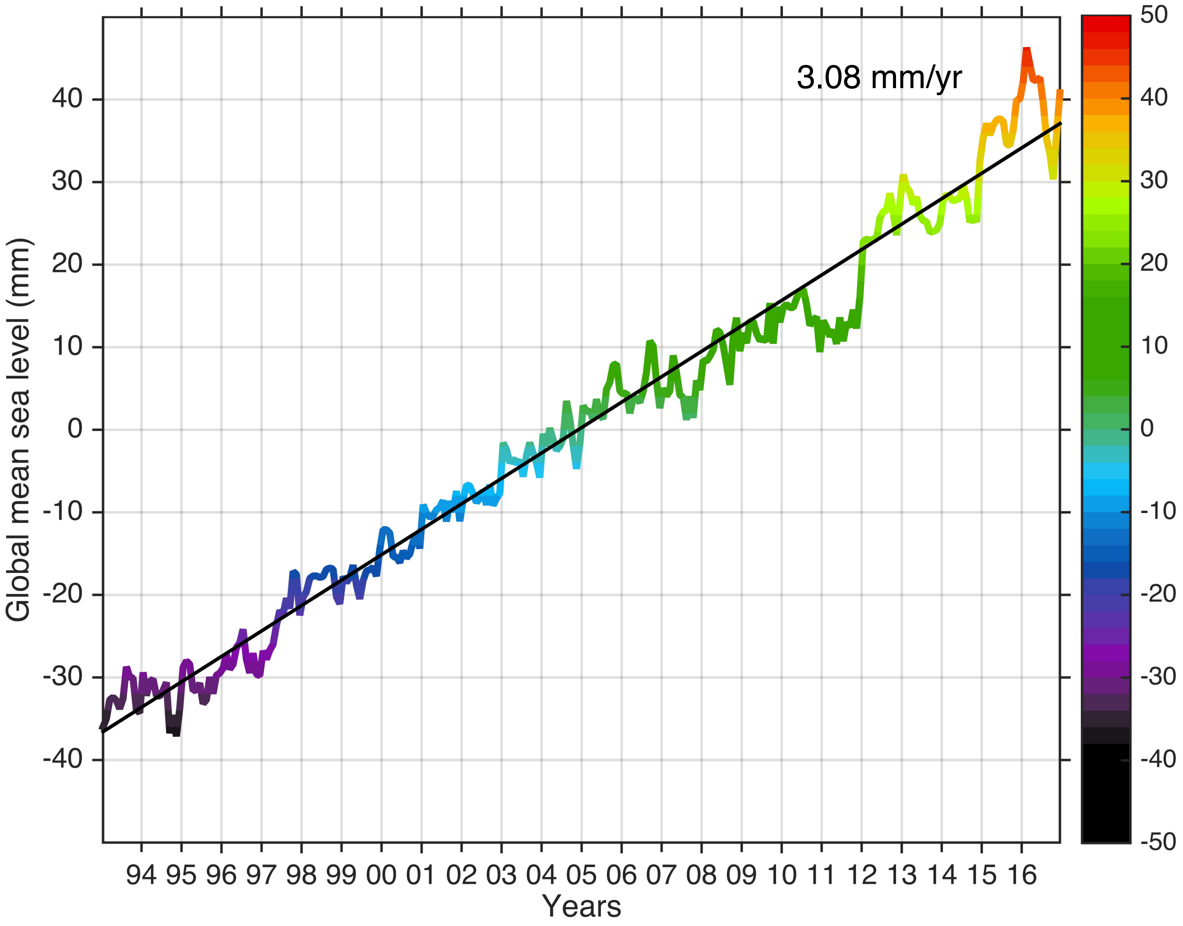

Figure S1: Global mean sea level curve. The deseasonalized gobal mean sea level curve from the multi-satellite altimetry missions calculated as the weighted average between 66S and 66N. The linear trend between 1993 and 2016 is estimated to about 3.1 mm/yr.

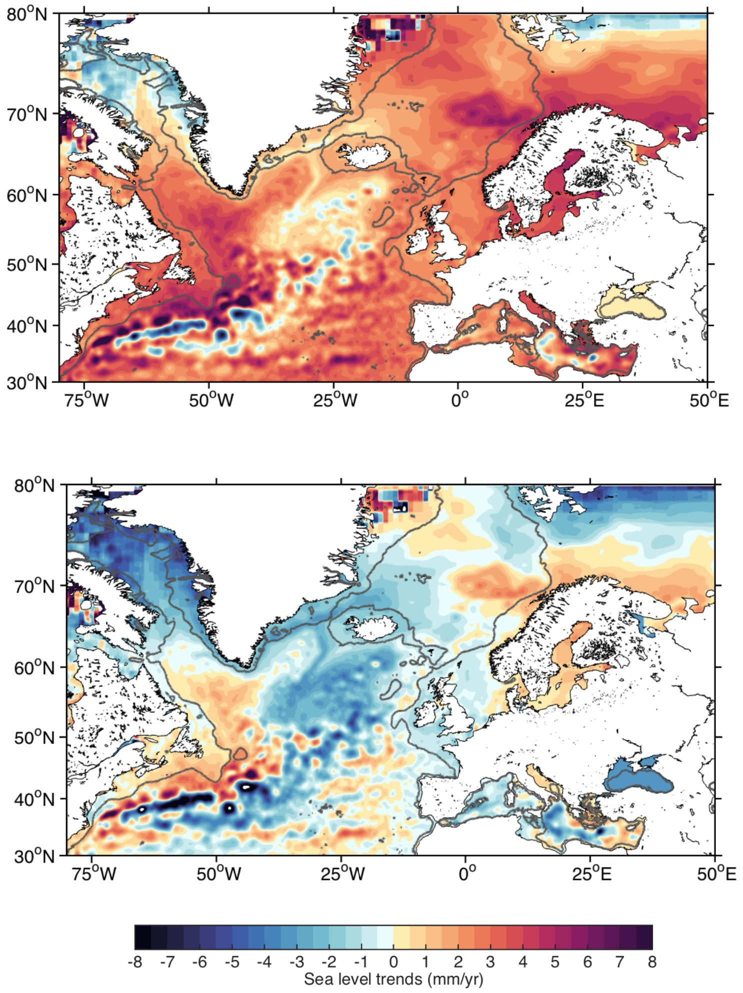

Figure S2: Long-term sea level trend. Spatial linear trend of sea level in the North Atlantic Ocean from satellite altimetry for the 1993-2016 period (upper) with and (lower) without global mean sea level trend shown in Fig. S1.

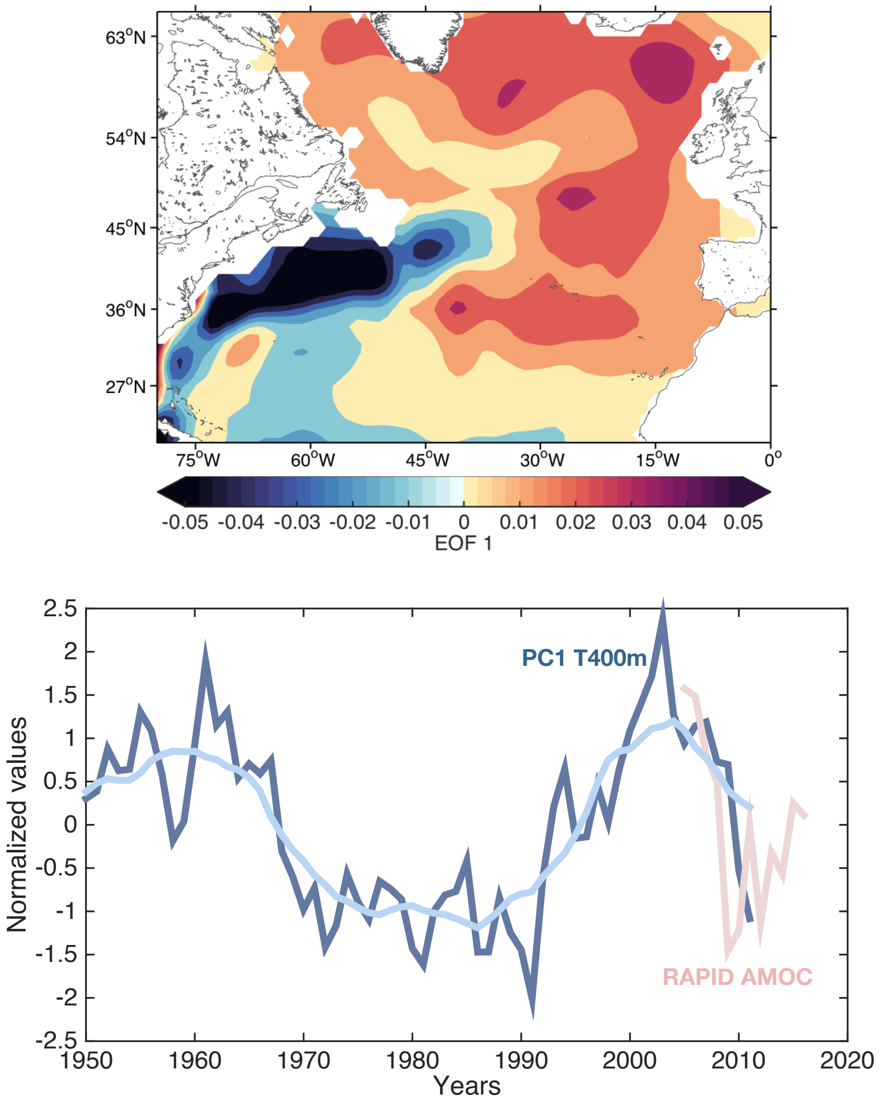

Figure S3: Spatio-temporal variability of the AMOC fingerprint. (upper) First leading mode of subsurface temperatures at 400 m depth from EN4 hydrographic analysis. (lower) The corresponding annual mean principal component (dark blue), its smoothed version using an 11-year running average (light blue) and normalized annual mean (based on monthly mean) RAPID AMOC index at 26ºN (Sv std^{-1}; light pink) for the 2005-2016 period (Smeed et al. 2017).

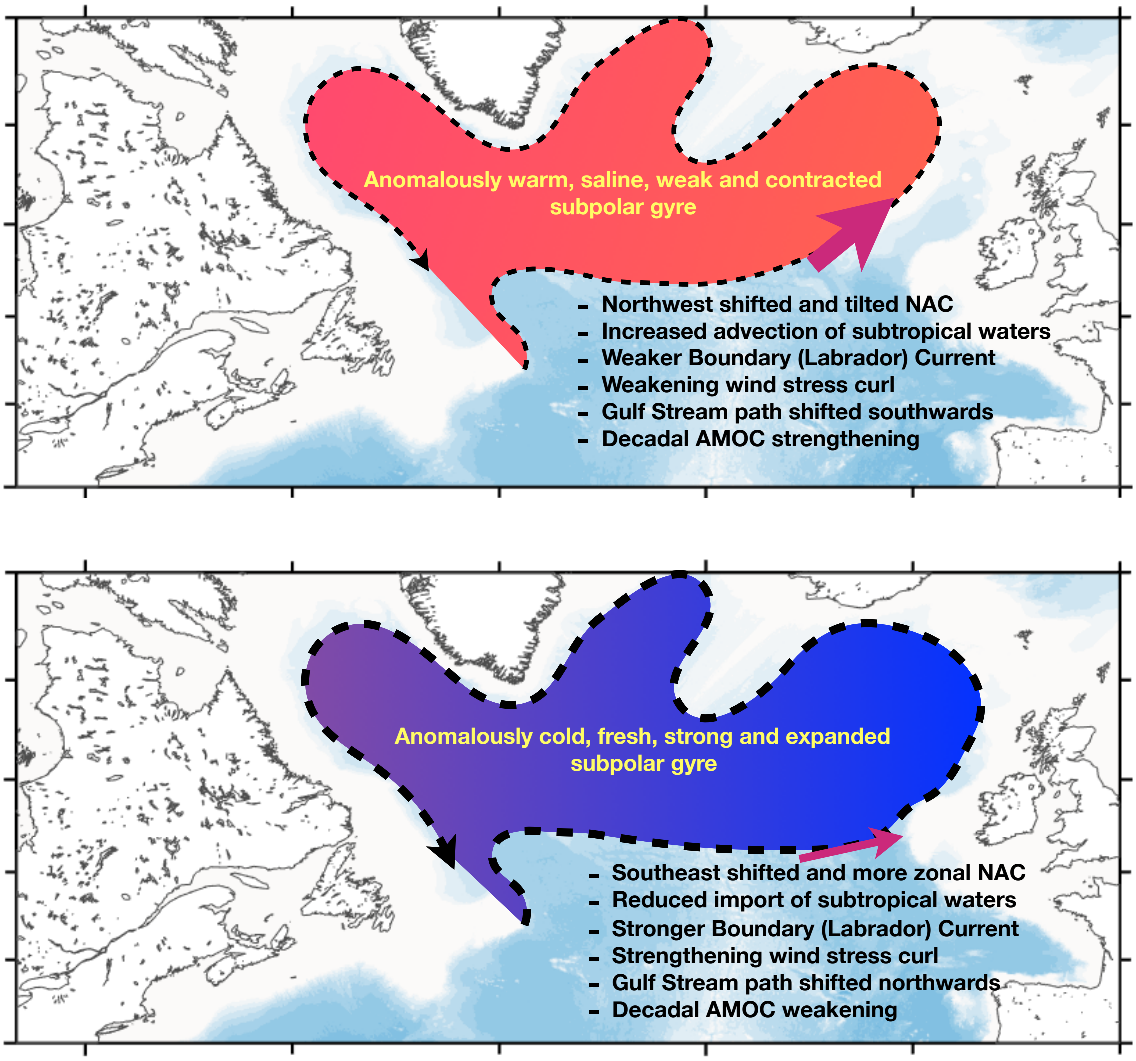

Figure S4: Schematic representation of the observed ocean circulation changes during the warming period (upper) and cooling period (lower) producing anomalously higher and lower SPNA sea level, respectively.

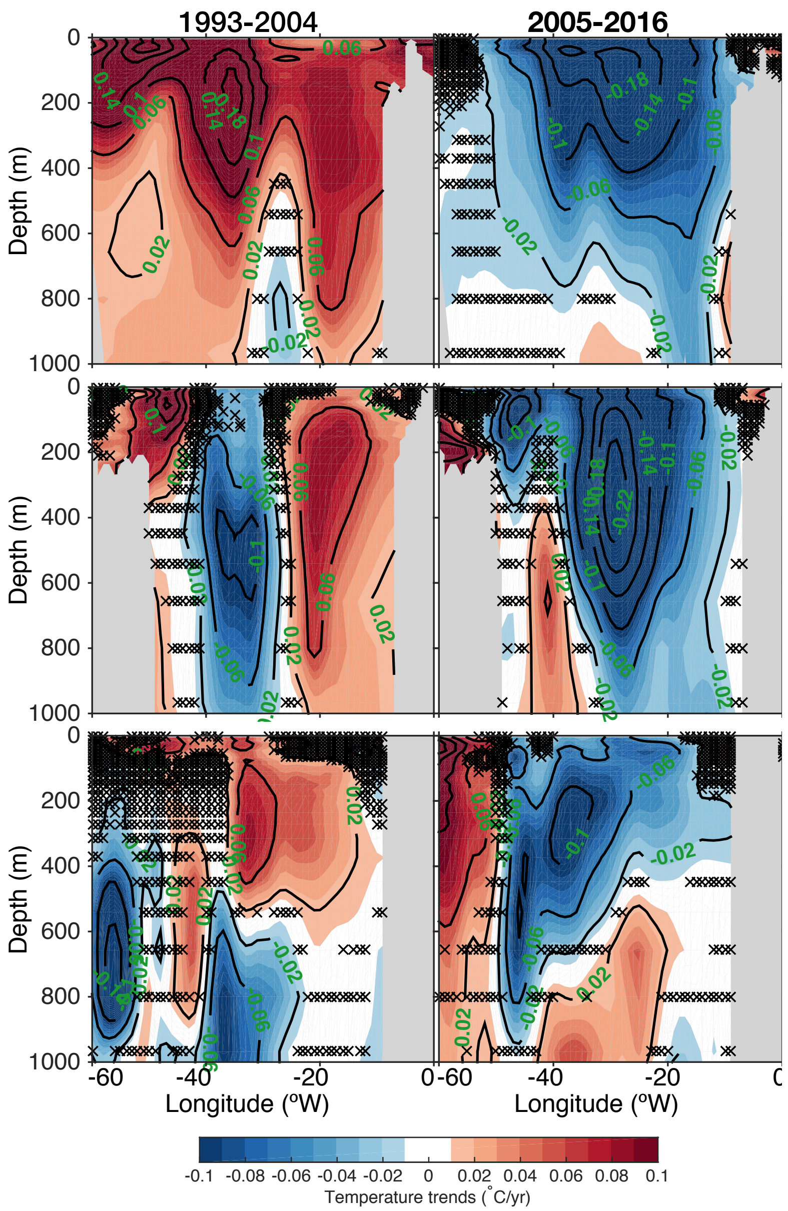

Figure S5. North Atlantic temperature trends. Depth-longitude temperature trends for the 1993-2004 (left column) and the 2005-2016 (right column) along the latitude band of 56N (upper row), 48N (middle row) and 40N (lower row). Note the spatially coherent temperature change at all latitudes, which in turn reflect the AMOC fingerprint pattern.

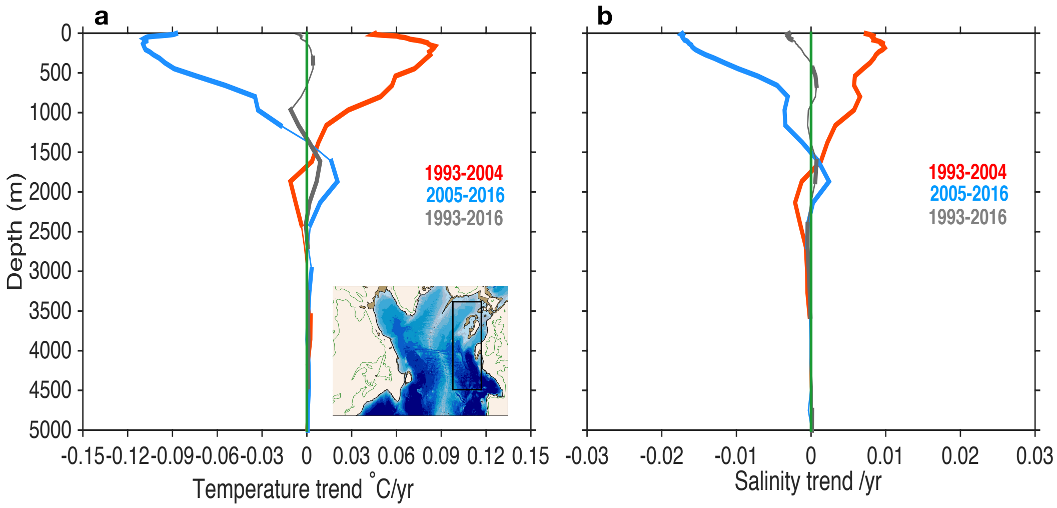

Figure S6. Temperature and salinity trends in the northeast Atlantic. (a) Temperature and (b) salinity trends per year for 1993-2004 period (red), 2005-2016 (blue) and the 1993-206 period (gray). The temperature and salinity are averaged in the northeast Atlantic (see the inset). Significant at the 95% confidence level are indicated by thicker lines. Warming (cooling) and salinification (freshening) for the 1993-2004 (2005-2016) suggest more (less) subtropical waters advected into the northeast Atlantic.

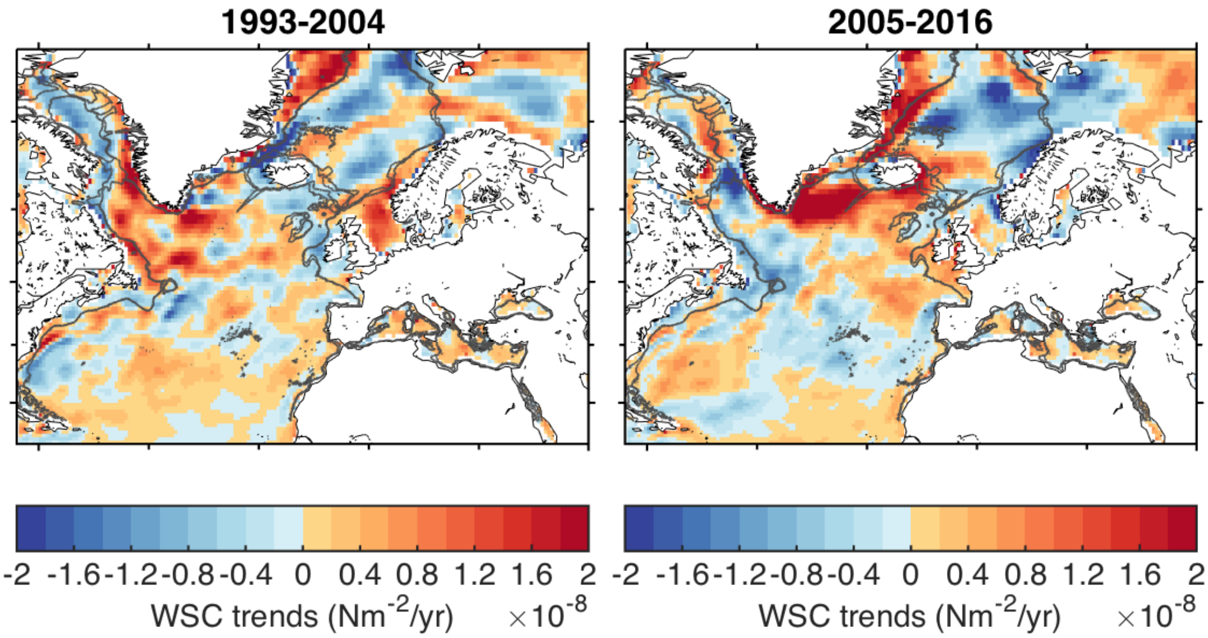

Figure S7: Wind stress curl trends based on ERA-interim reanalysis for the (left) 1993-2004 and (right) 2005-2016. The patterns are compatible with those derived using NCEP/NCAR reanalysis (Fig. 2).

Supplementary references

1. Smeed, D. et. al. Atlantic meridional overturning circulation observed by the RAPID-MOCHA-WBTS (RAPID-Meridional Overturning Circulation and Heatflux Array-Western Boundary Time Series) array at 26N from 2004 to 2017. Br. Ocean. Data Centre. doi: 10.5285/5acfd143-1104-7b58-e053-6c86abc0d94b (2017).
